# Trajectories of mental health problems in unaccompanied young refugees in Germany and the impact of post-migration factors – a longitudinal study

**DOI:** 10.1007/s00787-024-02535-2

**Published:** 2024-07-31

**Authors:** Fabienne Hornfeck, Maike Garbade, Selina Kappler, Rita Rosner, Elisa Pfeiffer, Cedric Sachser, Heinz Kindler

**Affiliations:** 1 German Youth Institute, Munich, Germany; 2 Department of Child and Adolescent Psychiatry/Psychotherapy, Ulm University, Ulm, Germany; 3 Department of Psychology, Catholic University Eichstätt-Ingolstadt, Eichstätt, Germany

**Keywords:** Unaccompanied young refugees, PTSS, Depression, Anxiety, Mental health, Post-migration factors

## Abstract

Unaccompanied young refugees (UYRs) are at an elevated risk of continuing psychological distress, including posttraumatic stress symptoms (PTSS), depression, and anxiety. The post-arrival situation in a host country plays an important role in increasing or reducing mental health risks for UYRs. This study aimed to examine the trajectory of psychological distress and post-migration factors experienced by UYRs in Germany with five assessments every six months over a two-year period.

Standardised self-report questionnaires were used to assess PTSS (CATS-2), depression (PHQ-9), and anxiety (GAD-7) symptoms. Material and social stressors (DSSYR), sociocultural adjustment (BSAS), frequency of contact with family members, and distress regarding asylum status were included as post-migration factors. *N* = 131 UYRs (M_age_ = 17.04; SD_age_ = 1.46; 81.1% male) living in residential care facilities in Germany were included in this study. There was a significant loss to follow up with *n* = 77 after 12 months at T2 (59% attrition), and *n* = 37 after 24 months at T4 (28% attrition).

At baseline, clinically relevant symptom scores were found in 48.1% for PTSS, 42.0% for depression, and 22.9% for anxiety. Analysis of latent growth curve models revealed that the symptom severity of depression and anxiety did not change substantially over one year, but PTSS showed a significant decline. The number of traumatic events affected initial levels of mental health problems but not their trajectory over time. The impact of post-migration factors varied between measurement points and outcome measures and did not result in a homogeneous pattern.

Despite limitations, this study demonstrated the persistence of high levels of psychological distress in UYRs. Effective interventions are required at an early stage to prevent chronic trajectories and support systems should adapt to constantly changing circumstances.

Trial registration number/date of registration: DRKS00017453/11th December 2019.

**Supplementary Information:**

The online version contains supplementary material available at 10.1007/s00787-024-02535-2.

## Introduction

Among the refugee population, unaccompanied young refugees (UYRs) are especially vulnerable to developing mental health problems. Compared to asylum-seeking and refugee children arriving together with at least one family member or relative, UYRs have a higher risk of developing serious mental health problems [1–4]. Review studies consistently reported that post-traumatic stress disorders (PTSD), depression, and anxiety are the most prevalent mental health problems in this group with up to 52% of UYRs scoring above the clinical cut-off for PTSD and up to 62% for internalising symptoms [5, 6]. Independent gender- and age-related differences in overall psychological symptoms between young refugees do not emerge clearly from the existing evidence as they may be influenced by confounding factors before, during, and after flight [7, 8]. The influence of traumatic experiences on UYRs’ level of mental health problems has been thoroughly studied, showing that the number and severity of stressful life events contribute to poor mental health outcomes [5, 6, 8]. Mental health problems of UYRs do not necessarily decrease after the arrival in a Western high-income country, which can sometimes be considered a “safe place”. While European states continue to discuss their reception capacities, refugees face ongoing challenges and stressors after their flights, especially when they are young and separated from their families [7]. In particular, stressors such as precarious living conditions, experiences of discrimination, financial worries, worries about family members, and fear of deportation represent risk factors for the mental health of refugees after they arrive in the host country [8–11] as they can impede coping with traumatic experiences, thereby increasing the incidence of mental disorders in refugees [12]. However, despite high levels of past and current adversity, UYRs often show considerable resilience and recover remarkably [13, 14]. Among other factors, social support, contact with family members, and successful acculturation have been proven to be protective in host societies [15–18]. Therefore, the post-arrival situation in the host country seems to play an important role in increasing or reducing mental health risks for vulnerable children and youth. The predominantly cross-sectional design of prior studies on this topic including published baseline results from this sample [19], makes it difficult to draw conclusions about the longitudinal course and reciprocal associations between the mental health problems of UYRs and post-migration factors.

A few longitudinal studies have investigated the trajectory of mental health outcomes in post-flight living conditions. In a Norwegian study of UYRs after their arrival in Norway, symptoms of depression, anxiety, and PTSS remained relatively stable at a high level over two years [10]. Persistently high symptom levels up to two years after arrival in the host country have also been found in UYR samples in Belgium [20] and in another Norwegian study [21] indicating a chronic trajectory of mental health problems. Jensen et al. [21] reported persistent patterns, especially among older UYRs. A substantial decline in symptom severity but still high levels of psychological distress after one year have also been found in asylum-seeking children and adolescents resettled in Germany [22]. In a cross-country study with UYRs on the move, anxiety, depression, and PTSS scores also decreased over two years [23, 24]. The same study also reported a decline in the number of daily stressors over time, while post-migration stressors (especially social stressors such as perceived discrimination or dissatisfaction with the educational situation) over 18 months after arrival increased in a UYR sample resettled in Belgium [20].

While findings from most cross-sectional studies indicate a clear association between post-migration stressors and mental health disorders, such as PTSS, depression and anxiety [5, 25], longitudinal studies are rare and findings are inconsistent. Vervliet et al. [20] reported that the number of perceived post-migration stressors led to significantly higher levels of PTSS, depression, and anxiety among UYRs in Belgium. Additionally, a recent two-year follow-up study of UYRs in Greece, Italy and Belgium showed that both, material and social stressors had a significant negative impact on anxiety and depression symptoms [24]. However, for PTSS, Pfeiffer et al. [23] found no significant effect of post-migration stressors over time in UYRs on the move. In addition to this cumulative analysis of post-migration stressors, some factors have been of special interest in previous cross-sectional studies. A positive influence on mental health has been found particularly for a secure asylum status [26], a higher frequency of contact with family members [17, 27], and there is evidence of a positive impact of successful sociocultural adaptation to the new environment [28].

Based on the results so far, no clear picture emerges about the longitudinal course of UYRs’ mental health problems after arrival in a host country. Further longitudinal studies are needed to examine reciprocal associations between pre- and post-migration stressors and mental health in these populations and how they change over time.

When conducting research with UYRs, particular challenges should be taken into account. Despite international standards aimed at ensuring UYR rights, migration policies and responsibilities for young migrants arriving without parents or legal guardians vary among countries. Different care systems and accommodation arrangements, ranging from low support facilities over UYR-specific residential group homes to foster families or independent apartments [29, 30], go along with distinct stressors and make it difficult to transfer findings from one context to the other.

### Research questions and hypotheses

The literature review of longitudinal studies demonstrates the complexity and ongoing challenges UYRs experience but also indicates huge research gaps in order to understand the post-migration process. The conduction of longitudinal studies including this population was consistently reported as a challenge for researchers and particularly high drop-out rates are common due to their high mobility when they are transferred within or across country borders or leave the care system [e.g., 10, 20–24]. The current study aimed to contribute to a better understanding of UYRs’ experiences after resettlement and therefore, examined the levels and trajectories of mental health problems in UYRs living in residential group homes in Germany and analysed the differential impact of post-migration factors on PTSS, depression, and anxiety. Baseline data from this study have previously been published [19]. To expand on this, this study aimed to answer the following research questions: To what extent do UYRs living in residential group homes report clinically relevant levels of depression, anxiety disorders, and PTSS? Based on previous research findings, prevalences of mental health outcomes in the clinical range are expected to be higher than in non-clinical samples of children and adolescents in Germany. The second question addresses changes in symptom levels over the two-year follow-up period and it is assumed that initially reported mental health problems persist over time. The third research question is concerned with the differential impact of post-migration factors on the mental health of UYRs and examines the following factors based on their high relevance in prior studies: material and social stressors, psychosocial adjustment, family contact, and distress regarding asylum status.

## Method

The study was approved by the ethics review board of the University Ulm (243/19) and Eichstaett-Ingolstadt (004–19). The BETTER CARE study was registered in the German Clinical Trials Register (www.germanctr.de; registration number DRKS00017453), and the study protocol was published before data collection [31].

### Procedure

Data were derived from the above-mentioned trial, and the longitudinal data of a predefined subsample of *N* = 131 UYRs at T0 were analysed. This study was conducted in residential care facilities for UYRs in four German states. After obtaining an agreement with the Children and Youth Welfare Services (CYWS) facilities, mental health screenings with UYRs were organised within the facilities. Interpreters were available in person or via phone if needed. Before the assessment, the UYRs were fully informed of the study’s objectives, procedures, and content. Inclusion criteria for participants were; (1) age 12–20 years at T0, (2) arrived in Germany as an unaccompanied minor, (3) applied for asylum or intended to do so, (4) being cared for by a CYWS facility, and (5) written informed consent provided by the participants and legal guardian (if < 16 years at baseline assessment (T0)), and (6) reported at least one traumatic event in line with the DSM-5 A criterion at T0 assessed via the traumatic event checklist of the CATS-2 at T0. Participants received compensation for taking part in assessments in the form of a voucher (€35 for each assessment). After T0 (*N* = 131), UYRs were screened again after six months (T1, *n* = 99; 75.6%), 12 months (T2, *n* = 77; 58.8%), 18 months (T3, *n* = 48; 36.6%), and 24 months (T4, *n* = 37; 28.1%). In order to reduce drop-out, the following measures were taken: face-to-face or online contact at every assessment, incentives for participation, individual assessments, high time flexibility of the study team regarding assessment planning, and motivation leaflets after T2 for follow-up assessments. The reasons for dropping out were most often loss of contact (*n* = 58) and lack of motivation and time (*n* = 23). The baseline recruitment and screening of UYRs occurred between July 2020 and July 2021 at 22 CYWS facilities. The outbreak of the COVID-19 pandemic in 2020 was accompanied by restrictions and consequences for everyday life in the following months and years and affected especially the first two waves of data collection. Assessments at all stages of the study were therefore performed via online tools or on-site, in compliance with strict hygiene standards.

### Sample

The initial sample consisted of *N* = 131 UYRs. 81.7% of the participants (*n* = 107) were male and one person (0.8%) indicated diverse genders. The age at baseline assessment ranged from 13 to 20 years (*M* = 17.04; *SD* = 1.46), and they had resided between 1 and 90 months in Germany (*M* = 25.75; *SD* = 20.52). At baseline, 32.8% of the UYRs reported an accepted asylum application signifying a permanent or temporary residence permit. The participants were from 29 countries of origin; most participants (*n* = 40; 30.5%) were born in Afghanistan.^1^

### Measures

The self-report questionnaires were available in German, English, French, Arabic, Dari, Farsi, Pashtu, Somali, Tigrinya, Russian and Kurmanci. Questionnaires were mainly completed on tablet computers via an online assessment tool. In case of technical problems or on request of participants, the questionnaires were also available on paper. In order to include participants with no or low reading abilities or varying dialects, interpreters were involved in the assessments. Demographic information assessed age, education, residential status, and living situation. Two items on distress and anxiety regarding current asylum status were rated on an 11-point Likert scale ranging from 0 to 10. Contact with family members was rated on a scale from 0 (*no contact*) to 5 (*daily contact*).

The *Child and Adolescent Trauma Screen (CATS-2)* [32] was used to assess PTSS in children and adolescents according to DSM-5 and ICD-11 criteria. In the current study, we used the traumatic event checklist to assess the number of potential traumatic events (PTEs) and DSM-5 total symptom score ranging from 0 to 60. A cut-off of 25 was set to indicate clinically relevant PTSS. Internal consistency (Cronbach’s α = 0.92 − 0.95) in our sample was found to be excellent.

The *Patient Health Questionnaire (PHQ-9)* [33] was used to measure depressive symptoms. Based on the validation study [29], scores of 10 and higher are classified as clinically relevant. The PHQ-9 has been validated in many contexts and languages [33, 34] and showed good reliability (Cronbach’s α = 0.83 – 0.89) in the current sample.

The *Generalized Anxiety Disorder Scale (GAD-7)* [35] is a 7-item rating scale based on the diagnostic criteria of the DSM-IV for generalised anxiety disorder. Scores of 10 or more indicate the presence of clinically relevant levels of anxiety. The GAD-7 has been validated in many contexts and languages [35]. In our sample, good reliability (Cronbach’s α = 0.81 – 0.95) was indicated.

The *Brief Sociocultural Adaptation Scale (BSAS)* [36] is a 12-item questionnaire that assesses various aspects of sociocultural adaptation in everyday life (e.g., language, climate, people, values and beliefs). The scale demonstrated acceptable reliability (Cronbach’s α = 0.79 – 0.87) and is available and validated in different languages. Data on this scale was missing in two cases in the current sample (1.5%).

The *Daily Stressors Scale for Young Refugees (DSSYR)* [37] assesses the extent to which participants have experienced 19 different post-migration daily stressors during the last month. Based on Behrendt et al. [24], seven items were clustered, indicating the number of social stressors, and nine formed material stressors. In two cases (1.5%), the mean scores could not be calculated due to too many missings. The subscales in our study showed at least acceptable reliability (Cronbach’s α material stressors = 0.76 – 0.84; Cronbach’s α social stressors = 0.65 – 0.85).

### Statistical analysis

**Fig. 1:**
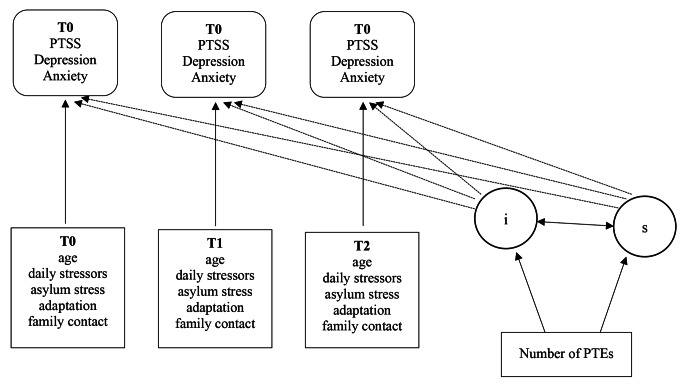
Illustration of conditional growth curve models of PTSS, depression, and anxiety in UYRs Note. *PTSS* Posttraumatic Stress Symptoms, *PTE* potential traumatic event, *i* intercept, *s* slope

Analyses were performed using IBM SPSS Statistics version 22 and R version 4.3.0 combined with the Lavaan package. According to Little’s Missing Completely at Random test, including all study variables, the missing data were not completely at random, *χ*^*2*^(391) = 443.964, *p* = .033. However, most of the missing data were due to wave nonresponse, particularly between waves T2 and T3. A binary logistic regression showed that attrition was not significantly related to sociodemographic characteristics (age and gender), mental health outcomes (CATS-2, PHQ-9, and GAD-7), or variables described as post-migration factors (DSSYR, BSAS, family contact, distress related to asylum status). Listwise deletion was applied to handle missing data. Descriptive statistics outlined the sociodemographic characteristics, means, standard deviations, and prevalence of all study variables. Latent growth curve models (LGCM) were applied to data from the first three time points only because of the extremely small sample size at T3 and T4 and the consequently limited feasibility of complex models. First, we calculated an unconditional growth model for the PTSS, depression, and anxiety scores to gain insight into the trajectories of mental health outcomes over time. The loadings for the slope factor were fixed at 0, 6, and 12, corresponding to the actual assessment time; therefore, the interpretation becomes for every *month* one increase in time. The variances for the three measurement points were fixed as equal, given the constant standard variations in mental health outcomes from T0 to T2 (see Table 1). Then, conditional growth models for each outcome variable (see Fig. 1) were built, including the time-invariant predictors of sex and the number of PTEs before arrival in the first step. In the second step, the time-varying covariates of age at assessment, material and social stressors, sociocultural adjustment, family contact, and distress regarding asylum status were included step-by-step into the model and then together in a final model. Indicators with a low model fit in the separate models were not included in the final model. For the model fit, different fit indices were used: the chi-square test statistic, Root Means Square Error of Approximation (RMSEA), and Comparative Fit Index (CFI). The cut-off criteria for the model fit indices are based on Hu and Bentler [38]. For the RMSEA, a value below 0.06 was considered a good fit, and a value below 0.08 was acceptable. For CFI, values above 0.95 were considered a good fit and values above 0.90 were acceptable. All tests were two-tailed, and an alpha significance level of α = 5% was used.

**Table 1:** Characteristics of participants and descriptive data of all study variables

	T0 (*n* = 131)	T1 (*n* = 99)	T2 (*n* = 77)	T3 (*n* = 48)	T4 (*n* = 37)
Gender (% male)	81.1	76.8	77.9	87.5	86.5
Age, *M (SD)*; range	17.04 (1.46); 13–20	17.54 (1.48); 14–21	17.96 (1.44); 14–21	18.24 (1.61); 15–22	18.66 (1.57); 16–22
Duration since arrival (months), *M (SD)*; range	25.75 (20.52); 1–90	29.73 (18.02); 7–78	33.30 (17.69); 13–84	35.69 (17.15); 19–90	40.92 (16.76); 25–95
Asylum application accepted (%)	32.8	38.3	36.3	50.0	44.8
Number of PTEs T0, *M (SD)*; range	6.56 (3.05); 1–14				
Distress regarding asylum status, *M (SD)*; range	5.60 (3.48); 0–10	5.32 (3.52); 0–10	5.36 (3.47); 0–10	4.83 (3.26); 0–10	4.72 (3.25); 0–10
Family contact, *M (SD)* *0 - No contact (%)* *1 - Once a year or less (%)* *2 - Several times a year (%)* *3 - Monthly (%)* *4 - Weekly (%)* *5 - Daily (%)*	2.27 (1.96); 0–5 35.1 6.9 6.9 12.2 24.4 14.5	2.41 (1.96); 0–5 34.0 4.3 5.3 13.8 27.7 14.9	2.46 (1.95); 0–5 33.8 2.5 2.5 22.5 22.5 16.3	1.81 (1.97); 0–5 47.2 5.6 2.8 25.0 2.8 16.7	2.28 (1.94); 0–5 37.9 0 6.9 17.2 27.6 10.3
BSAS, *M (SD)*; range	4.90 (0.91); 1.50–6.92	4.84 (0.86); 2.50–6.33	4.80 (0.88); 2.17–6.33	4.99 (0.92); 3.08–6.92	4.96 (0.95); 2.75–6.33
DSSYR material, *M (SD)*; range	3.62 (2.48); 0–9	3.62 (2.73); 0–9	3.74 (2.49); 0–9	3.03 (2.64); 0–9	3.07 (2.62); 0–8
DSSYR social, *M (SD)*; range	4.05 (1.83); 0–7	4.26 (2.20); 0–7	4.18 (2.15); 0–7	4.05 (2.08); 0–7	3.79 (2.36); 0–7
CATS-2, *M (SD)*; range	24.56 (11.46); 1–56	23.12 (12.02); 0–56	21.19 (10.93); 0–54	22.76 (11.10); 0–43	22.65 (12.66); 0–50
PHQ-9, *M (SD)*; range	8.69 (5.55); 0–24	8.36 (5.39); 0–24	8.02 (5.71); 0–25	7.27 (5.73); 0–18	7.83 (5.58); 0–19
GAD-7, *M (SD)*; range	7.10 (4.80); 0–19	6.17 (4.87); 0–20	5.96 (4.74); 0–19	5.78 (4.18); 0–20	6.90 (5.69); 0–21

*Note*. *M* Mean, *SD* Standard Deviation, *PTE* potential traumatic event, *DSSYR* Daily Stressors Scale for Young Refugees, *BSAS* Brief Sociocultural Adaptation Scale, *CATS-2* Child and Adolescent Trauma Screen 2, *PHQ-9* Patient Health Questionnaire-9, *GAD-7* Generalized Anxiety Disorder Scale-7

## Results

The descriptive characteristics of all study variables are presented in Table 1.

**Fig. 2:**
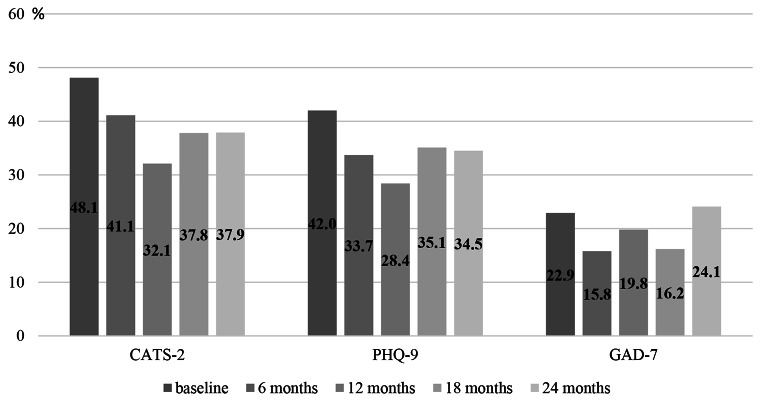
Frequency of sum scores above the cut-off for CATS-2, PHQ-9, and GAD-7 *Note*. *n* = 37–131, *CATS-2* Child and Adolescent Trauma Screen 2, *PHQ-9* Patient Health Questionnaire-9, *GAD-7* Generalized Anxiety Disorder Scale-7

Further analyses regarding symptom scores of the included mental health outcomes revealed that between 32.1% (*n* = 26; T2) and 48.1% (*n* = 63; T0) of UYRs showed clinically relevant levels of PTSS, the frequency for elevated levels of depressive symptoms ranged between 28.4% (*n* = 23; T2) and 42.0% (*n* = 55; T0), and regarding anxiety symptoms, between 15.8% (*n* = 15; T1) and 24.1% (*n* = 7; T4) of UYRs scored in the clinical range (see Fig. 2 and supplementary material for comparison to ICD-11 criteria). 19.1% (*n* = 25) of UYRs showed elevated levels in all three domains at baseline, 14.7% (*n* = 14) after 6 months, 14.8% (*n* = 12) after 12 months, 13.5% (*n* = 5) after 18 months, and 17.2% (*n* = 5) at 24-months-follow up.

### Unconditional growth models

First, for PTSS, the linear trajectories provided a good fit to the observed data, *χ*^*2*^(3) = 3.801, *p* = .284, *CFI* = 0.992, *RMSEA* = 0.061, *CI*_*RMSEA*_ = 0.000 – 0.217. The mean starting point of PTSS at baseline was estimated to be 24.560 (*SE* = 0.1.471), *p* < .001, and the mean of the slope was − 0.273 (*SE* = 0.107), *p* = .010 indicating a significant decrease in PTSS over time. Second, for depressive symptoms, fit indexes indicated a very good model fit to the observed data, χ^2^(3) = 1.440, *p* = .696, *CFI* = 1.000, *RMSEA* = 0.000, *CI*_*RMSEA*_ = 0.000 – 0.149. The mean starting point of depressive symptoms at baseline was estimated to be 8.444 (*SE* = 0.653), *p* < .001), and the mean of the slope was − 0.056 (*SE* = 0.058), *p* = .336. Thus, depressive symptoms remained stable over time. The third model for anxiety symptoms also fits the data well, *χ*^*2*^(3) = 2.416, *p* = .491, *CFI* = 1.000, *RMSEA* = 0.000, *CI*_*RMSEA*_ = 0.000 – 0.183. The mean starting point of anxiety symptoms at baseline was estimated to be 6.789 (*SE* = 0.553), *p* < .001, and the mean of the slope was − 0.087 (*SE* = 0.053), *p* = .102. Thus, anxiety symptoms showed no significant changes over time.

### Conditional growth models

Table 2 presents the results of the final models for all three mental health outcomes. The final model for ***PTSS*** reached a reasonable model fit, *χ*^*2*^(41) = 56.562, *p* = .054, *CFI* = 0.911, *RMSEA* = 0.082, *CI*_*RMSEA*_ = 0.000 – 0.130. Regarding the number of pre-migratory PTEs, a significant effect was found for the intercept; however, this factor did not affect the slope. A significant association was found between higher PTSS scores and more distress related to asylum status at T0, more social stressors at T0, and less contact with family members at T1. The final model for ***depression*** resulted in a good model fit, *χ*^*2*^(41) = 46.736, *p* = .249, *CFI* = 0.955, *RMSEA* = 0.050, *CI*_*RMSEA*_ = 0.000 – 0.107. The number of PTEs had a significant effect on the level of depressive symptoms at baseline, with a higher number of PTEs predicting a higher symptom score; however, they did not affect the longitudinal trajectory. The levels of sociocultural adjustment and social stressors were also significantly related only to depressive symptoms at baseline indicating that lower levels of adjustment and more social stressors were associated with higher symptom scores at T0. Moreover, a higher number of material stressors were significantly associated with a higher score for depressive symptoms at T1.

**Table 2:** Standardised estimates and standard errors of time-invariant and time-variant factors as the result of conditional growth curve models of PTSS, depression, and anxiety in UYRs.

	PTSS, *n* = 57						Depression, *n* = 57						Anxiety, *n* = 57					
	Intercept			Slope			Intercept			Slope			Intercept			Slope		
	Estimate	SE	*p*	Estimate	SE	*p*	Estimate	SE	*p*	Estimate	SE	*p*	Estimate	SE	*p*	Estimate	SE	*p*
Gender	0.25	2.25	0.910	− 0.07	0.22	0.766	0.81	1.18	0.494	− 0.10	0.14	0.457	0.32	1.04	0.760	− 0.15	0.12	0.224
PTEs	**1.83**	**0.37**	0.000***	− 0.05	0.04	0.182	**0.62**	**0.20**	0.002**	0.01	0.02	0.611	**0.64**	**0.16**	0.000***	− 0.0	0.02	0.646
	Regressions						Regressions						Regressions					
	Estimate	SE	*p*				Estimate	SE	*p*				Estimate	SE	*p*			
Age at assessment T0	− 0.16	0.66	0.805				− 0.05	0.36	0.888				**− 0.26**	**0.31**	**0.405**			
Age at assessment T1	1.22	0.71	0.084				0.26	0.31	0.395				0.02	0.28	0.938			
Age at assessment T2	0.45	0.87	0.603				0.23	0.40	0.568				0.35	0.36	0.328			
Distress regarding the asylum status T0	**1.01**	**0.29**	0.000***				0.11	0.17	0.525									
Distress regarding the asylum status T1	0.13	0.24	0.585				− 0.13	0.12	0.300									
Distress regarding the asylum status T2	0.17	0.31	0.596				0.25	0.17	0.142									
Frequency of family contact T0	0.14	0.52	0.784				0.26	0.30	0.374				− 0.19	0.26	0.461			
Frequency of family contact T1	**-1.21**	**0.58**	0.037*				− 0.34	0.27	0.219				− 0.37	0.25	0.139			
Frequency of family contact T2	− 0.52	0.69	0.452				− 0.15	0.33	0.660				− 0.31	0.30	0.312			
Sociocultural adaptation T0	0.17	1.07	0.870				**-1.82**	**0.63**	0.004**				− 0.49	0.58	0.394			
Sociocultural adaptation T1	-2.25	1.20	0.061				− 0.75	0.60	0.208				− 0.59	0.59	0.316			
Sociocultural adaptation T2	-1.09	1.37	0.427				− 0.32	0.74	0.669				-1.12	0.68	0.102			
Social stressors T0	**18.05**	**4.04**	0.000***				**5.93**	**2.42**	0.014**				**6.14**	**2.18**	0.005**			
Social stressors T1	1.55	3.27	0.636				0.31	1.61	0.848				1.86	1.60	0.245			
Social stressors T2	0.82	4.32	0.849				4.41	2.35	0.060				2.17	2.19	0.322			
Material stressors T0	− 0.96	3.32	0.772				1.82	2.01	0.366				0.16	1.78	0.930			
Material stressors T1	1.72	3.12	0.583				**4.02**	**1.55**	0.010*				1.69	1.55	0.278			
Material stressors T2	5.53	4.35	0.203				0.89	2.39	0.708				− 0.10	2.20	0.962			

*Note*. *SE* standard error, *p < .05, **p < .01, and ***p < .001. PTE potential traumatic event

In the final model of ***anxiety***, distress regarding asylum status was excluded because of a failed model fit. The final model including all remaining factors, had an acceptable model fit, *χ*^*2*^(35) = 43.921, *p* = .143, *CFI* = 0.911, *RMSEA* = 0.067, *CI*_*RMSEA*_ = 0.000 − 0.123. A significant impact on the baseline level was found for the number of PTEs, indicating that a higher number of PTEs was associated with more anxiety symptoms. Furthermore, younger age and higher levels of social stressors were associated with higher levels of anxiety symptoms at T0.

## Discussion

This study focused on the mental health of UYRs living in residential care facilities in Germany at the beginning of the study and aimed to contribute to a better understanding of the longitudinal mental health trajectories and the role of risk and protective factors after arrival in a European host country. Baseline assessment occurred 1–90 months after arrival in Germany with a mean period of nearly two years in Germany at baseline assessment. Four follow-up assessments every six months were conducted. Like in most high-income countries, unaccompanied children and youth arriving in Germany under the age of 18 have access to specific services and support within the Children and Youth Welfare System. The results of the current prospective longitudinal study confirmed the findings gathered from recent reviews [5, 6] indicating that UYRs are a highly vulnerable group with a high prevalence of PTSS (32.1 − 48.1%), depression (28.4 − 42.0%), and anxiety (15.8 − 24.1%) symptom scores above the clinical cut-off persisting in a post-flight phase of 24 months of follow-up after initial assessment. However, compared to youth living in refugee camps in Europe or low-income countries, a review by Vossoughi and her colleagues reported even higher but extremely varying incidences of mental health problems within this population with up to over 90% exhibiting severe mental health problems, e.g., PTSD, depression and anxiety symptoms [39].

Regarding the trajectory of symptom severity, the findings showed that anxiety and depressive symptom scores did not significantly change over time and, on average, remained at a subclinical level through the first years after resettlement in the host country. This chronicity of psychological distress has also been demonstrated in studies on UYRs living in other European countries [10, 20, 21] and might be explained by ongoing or newly emerged stressors in post-flight situations (e.g., fear of the future, and dissatisfaction in resettlement circumstances). Conversely, consistent with prior studies [22–24, 40], PTSS of UYRs in this sample tended to decline slightly but significantly in a 12-months-period indicating that UYRs might recover to some extent from their pre-arrival and flight-related traumatic experiences after arrival in a safer place, especially regarding the symptoms of hyperarousal. However, the descriptive data of mental health outcomes at the 18- and 24-month follow-ups show a tendency toward a renewed increase in symptom levels. A possible reason for this can be seen in the potential stress factors associated with the age of the majority of the remaining sample, as the mean age at T3 was approximately 18 years. For UYRs, turning 18 may be associated with a realistic fear of leaving the youth welfare system or even possible deportation [7]. Furthermore, the results must also be interpreted in light of the COVID-19 pandemic since worries related to the disease and restrictions, such as school closures, affected the mental health of children and youth in general and led to an increase in mental health problems in the migrant population during this period [41]. Therefore, it cannot be ruled out that the pandemic distorted our findings and led to variations in symptom levels over this 2-year period of significant social disruptions, isolation and uncertainty about the future.

The analysis of potential predictors of mental health problems is highly heterogeneous and varies greatly between the outcomes and measurement points. Gender was the only exception, with no significant impact on the baseline level or slope of mental health outcomes. This is in contrast to most studies including UYR samples, indicating that female gender and older age were associated with poorer mental health outcomes [25]. However, this result needs to be interpreted with caution due to the unequal distribution of this variable and a typically lower number of girls among the UYR population. While the number of pre-migration traumatic experiences affected the severity of UYRs’ mental health problems at the beginning of the study, no ongoing impact of trauma load on mental health outcomes was found, indicating that the well-being of UYRs might depend more on situational conditions and stressors in the “here and now” than on pre-migration trauma. This is surprising, given the empirical evidence of the enormous ongoing relevance of the severity and number of presettlement traumatic events found in refugee studies focusing on psychological stress [6, 17]. One explanation could be the stabilising effect of living conditions and support within the youth welfare system, making it easier to establish an emotional distance from past traumatic experiences [13, 14]. However, accepting or avoiding these experiences may have also reduced their impact over time [42]. Furthermore, remission from PTSS was also found to be common within a mean of three years after the first assessment in clinical samples [43]. With regard to the high variance of how long the participants of the current study have been already living in Germany, this might be an important mechanism for those children and youth with a longer stay. Conversely, more time does not necessarily mean more stability and permanence: facilities should guarantee a minimum living standard, but the quality between facilities might be as diverse as the needs of every individual and care policies and practices differ extremely even on a regional level [29, 30]. Consequently, post-migration stressors have been shown to have greater relevance in UYRs’ everyday lives over time and might supersede the effect of traumatic experiences [43, 44]. In the present study, all factors included in the post-migration context (material and social stressors, psychosocial adjustment, family contact, and distress regarding asylum status) had a meaningful impact on mental health and this effect was most evident at the beginning of the study at baseline assessment but not in the longitudinal trajectory. Furthermore, a wide range of potentially crucial individual and situational circumstances may not have been captured within this study. Specific pandemic-related circumstances, leaving the care system, school graduation, political and security changes or deterioration of the security situation in the home country (especially with regard to the large group of Afghan UYRs within the sample (28 − 38%)) can contribute to new uncertainties and higher psychopathology for adolescents in general and UYRs specifically. These factors may have an enormous impact on the individual level and should be addressed in future studies.

### Strengths and limitations

As a strength, the study used validated and standardised self-report measures to assess mental health outcomes and post-migration stressors in UYRs, which allows for comparison of the study results with the findings of previous and future studies. Additionally, the study sample was highly heterogeneous, including UYRs from different countries of origin and geographical regions, and screening was performed in 22 different CYWS facilities all over Germany. Moreover, different post-migration factors (e.g. daily stressors and sociocultural adaptation) were considered in the analyses. Furthermore, data were assessed longitudinally, and despite the enormous mobility of the population, in a substantial number of cases, at least three measurement points within one year were realised.

However, this study has several limitations. First, the limited number of participants and the high proportion of dropouts, especially at T3 and T4, restricted the feasibility of several analyses. Despite intensive efforts to reduce drop-out, reaching the participants especially 18 and 24 months after the initial assessment remained a major challenge of the study and resulted in a high amount of missing values. Maintaining contact with the involved UYRs between the assessments was particularly difficult when they left the facility (for diverse reasons) and the study team heavily relied on the commitment of social workers to follow them up. However, the attrition within the current sample after 12 months was comparable to the longitudinal assessment in the study of Jakobsen and colleagues [10]. Second, our results may have been influenced by selection bias, as participation in the study was voluntary, and UYRs with severe or no mental health problems may not have participated. Therefore, these results may not be fully generalisable to the UYR population resettled in Germany. Third, social desirability may have influenced the responses of the UYRs, resulting in an underestimation of the effects. Fourth, despite the high validity of the questionnaires for mental health outcomes, the screening instruments for PTSS, depression, and anxiety are not sufficient to obtain valid diagnoses, as screening self-report measures tend to result in a slightly higher prevalence compared to interviews [45]. In future studies, more detailed information should be obtained using semi-structured interviews to determine symptom loads and diagnoses of PTSD, depression and anxiety. Caution is also warranted when interpreting the results of the DSSYR questionnaire and the two-factor structure because broad validation is still lacking, and the reliability was barely acceptable at some of the measurement points in the current study; however, the questionnaire has already been widely used in the field of refugee mental health [21, 23, 24]. Fifth, regarding the variety of geographical regions of origin, the analyses did not allow to distinguish between countries of origin due to the small group sizes; therefore, the results might not be comparable to studies including only war-affected participants [46]. Sixth, given the high proportion of male participants (81.7%), the results cannot be easily transferred to merely female groups. However, a relatively small proportion of girls is representative of the group of UYRs in Germany [47] and UYR samples in most international studies conducted in European countries [8]. Seventh, the UYR’s physical health status and current use of (mental) health services were not included in the analysis because of a lack of detailed data provided on this information. Eighth, the results must be interpreted cautiously because of the small sample size, limiting their generalisability. In particular, there is a tendency to over-reject structural equation models in the case of small sample sizes (*N* < 250) [38]. Ninth, the findings within this population cannot be generalised to even more neglected subpopulations, like e.g., UYRs living in reception centres for adults and do therefore not receive adequate care and services despite their young age. Finally, another limitation arises from the assessments conducted under pandemic conditions; therefore, the results cannot be easily transferred to other conditions.

## Implications and conclusions

This longitudinal study contributes to the current knowledge about UYRs’ mental health and the complex nature of the refugee experience in the first years after arrival in a host country. The findings indicate, that after placement within the care system in Germany, UYRs still suffer from high levels of psychological distress and trauma-related symptoms. These results demonstrate the ongoing impact of past traumatic experiences on the one hand and the constant effect of current post-migration stressors on UYRs’ mental health on the other hand. The results emphasise the high need for early mental health screening and a treatment with empirically supported mental health interventions in order to reduce symptoms of PTSD, depression, and anxiety. It is of utmost importance to prevent chronic courses by reducing post-migration stressors at an early stage after arrival. Despite the evident mental health burden, UYRs encounter barriers in assessing the treatment they need and policymakers in European countries fail to prioritise the protection and support of the most vulnerable groups and refrain from investing in improving regional mental health and care systems. The findings also point to the need to consider mental health issues in light of the current reality of the UYRs’ lives with a focus on fluctuating individual and contextual conditions over time. Most UYRs are subject to various transitions across services and systems, e.g., when they turn 18 or when they receive a decision about their asylum application [29, 30]. Future studies should consider mixed-method approaches to achieve a systematic understanding of the complexity of factors that increase or reduce mental health risks for UYRs after arrival in a host country in order to provide adequate and sustained support. More evidence is also needed about the trajectories of mental health problems in UYRs and the impact of post-migration factors over time, especially when UYRs reach adulthood and leave a safe care system.

## Electronic supplementary material

Below is the link to the electronic supplementary material.

Supplementary Material 1

## Data Availability

The datasets generated for this study are available from the corresponding author on request.

## Footnotes

1 For a more detailed description of the sample and included questionnaires see 27.
