# Supplementary material for: Trajectories of mental health problems in unaccompanied young refugees in Germany and the impact of post-migration factors – a longitudinal study
Source: Eur Child Adolesc Psychiatry. 2024 Jul 31;34(3):1051–62. doi: 10.1007/s00787-024-02535-2 (PMC11909058; doi:10.1007/s00787-024-02535-2)
Supplement: Supplementary file 1 — Supplementary Material 1 [file 787_2024_2535_MOESM1_ESM.docx]

**Table X.** Descriptive data of CATS-2, analyzed according to DSM-5 or ICD-11.

|  | Baseline (*n*=131) | 6 months  *(n*= 95) | 12 months  (*n*=81) | 18 months  (*n*= 37) | 24 months  (*n*= 29) |
| --- | --- | --- | --- | --- | --- |
| CATS-2 DSM-5  PTSD Sum score | 24.56  (11.46) | 23.12 (12.02) | 21.19 (10.93) | 22.76 (11.10) | 22.66 (12.66) |
| CATS-2 ICD-11  PTSD Sum score | 8.23  (4.00) | 7.64  (3.84) | 6.74  (3.70) | 7.62  (3.98) | 8.03  (4.40) |
| CATS-2, ICD-11  CPTSD Sum score | 14.31  (7.23) | 13.57  (7.44) | 12.38  (6.70) | 13.08  (7.14) | 13.59  (7.64) |

*Note. n* = 29-131.

**Table X.** *Frequency of sum scores above the cut-off for CATS-2 according to ICD-11.*

|  | Baseline (*n*=131) | 6 months  *(n*= 95) | 12 months  (*n*=81) | 18 months  (*n*= 37) | 24 months  (*n*= 29) |
| --- | --- | --- | --- | --- | --- |
| CATS-2 ICD-11 PTSD or CPTSD | 60 (45.8) | 40 (42.1) | 23 (28.4) | 15 (40.5) | 12 (41.4) |
| CATS-2 ICD-11 PTSD | 24 (10.7) | 11 (11.6) | 4 (4.9) | 3 (8.1) | 3 (10.4) |
| CATS-2 ICD-11 CPTSD | 46 (35.1) | 29 (30.5) | 19 (23.5) | 12 (32.4) | 9 (31.0) |

*Note. n*(%), *CATS-2* Child and Adolescent Trauma Screen 2.

**Figure X**

*Frequency of sum scores above the cut-off for CATS-2 according to ICD-11*

*Note.* *CATS-2* Child and Adolescent Trauma Screen 2, values are percentages.
